# Association of Early Feeding Practices with Gastrointestinal Symptoms in Infants During the First 12 Months: A Multicenter Prospective Cohort Study

**DOI:** 10.3390/nu18091383

**Published:** 2026-04-28

**Authors:** Yaxin Yu, Jiahui Zhang, Xinyue Wang, Simin Zhang, Yuluyuan Tian, Xianfeng Zhao, Shuangling Sun, Zhixu Wang, Xiaoqin Luo

**Affiliations:** 1 Department of Nutrition and Food Safety, School of Public Health, Xi’an Jiaotong University, Xi’an 710061, China; y1144636511@stu.xjtu.edu.cn (Y.Y.);; 2 Danone Open Science Research Center, Shanghai 201204, China; xianfeng.zhao@danone.com (X.Z.); lynn.sun@danone.com (S.S.); 3 Department of Maternal, Child and Adolescent Health, School of Public Health, Nanjing Medical University, Nanjing 211166, China; 4 Key Laboratory for Disease Prevention and Control and Health Promotion of Shaanxi Province, Xi’an 710061, China

**Keywords:** feeding practices, exclusive breastfeeding, IGSQ, gastrointestinal symptoms

## Abstract

Background: Functional gastrointestinal disorders (FGIDs) are highly prevalent among infants. Exclusive breastfeeding has been consistently associated with better gastrointestinal health. However, current evidence regarding the associations between early feeding practices and infant gastrointestinal development remains limited. Objectives: To examine the associations between early feeding practices at 1 month of age and gastrointestinal symptoms and overall gastrointestinal burden in infants during the first 12 months of life. Methods: In this multicenter prospective cohort study, 669 healthy mother–infant pairs were finally included. According to feeding practices at 1 month of age, infants were categorized into three groups: exclusive direct breastfeeding (EDB, *n* = 236, 35.28%), bottle-fed expressed breastmilk (EBB, *n* = 150, 22.42%), and mixed feeding (MF, *n* = 283, 42.30%). Gastrointestinal (GI) symptoms were assessed using the Infant Gastrointestinal Symptom Questionnaire (IGSQ) and symptom items from the PedsQL™ Infant Scales. Generalized estimating equations (GEEs) were used to assess the associations. Results: Infants in the EDB group had the lowest incidence of GI symptoms and lower IGSQ scores throughout the follow-up period. Compared with EDB, the MF group showed higher IGSQ scores (β = 0.95, *p* = 0.002) and higher odds of constipation (OR = 1.64, *p* < 0.001), vomiting (OR = 1.70, *p* < 0.001), and swallowing difficulty (OR = 1.79, *p* = 0.002); these associations remained robust across multiple sensitivity analyses. The EBB group showed higher odds of certain symptoms in the main analysis, but sensitivity analyses (e.g., time-varying exposure) indicated that these associations were not robust, except for bloating (OR = 1.31, *p* = 0.042). Conclusions: The EDB is the optimal strategy for infant gastrointestinal health and should be prioritized. The MF is robustly associated with increased odds of constipation, vomiting, swallowing difficulty, and overall gastrointestinal burden. The EBB may slightly increase the odds of bloating, which can be mitigated by paced feeding and adequate burping.

## 1. Introduction

Infancy represents a critical window for the development and functional maturation of the gastrointestinal (GI) tract, during which GI discomfort is relatively common [1]. Functional gastrointestinal disorders (FGIDs) in infants are typically associated with immature GI development or poor adaptation to internal and external stimuli. Although symptoms often resolve with age, FGIDs can significantly affect infant health, sleep quality, and emotional well-being in early life, increase the burden of caregiving on families [2], and potentially exert lasting negative effects on long-term health outcomes [3,4].

Epidemiological evidence indicated that the overall prevalence of FGIDs in Chinese infants is approximately 27.3%, with symptoms, such as colic and reflux, being more prevalent than in Western countries [3,5,6,7]. Among the modifiable factors, feeding practices are believed to play a pivotal role in shaping GI development. Evidence suggests that exclusive direct breastfeeding not only reduces the risk of FGIDs, but also supports gastrointestinal homeostasis by modulating the gut microbiota, promoting intestinal motility, and maintaining mucosal barrier integrity [8,9,10]. Furthermore, prolonged breastfeeding may enhance these protective effects [11]. However, systematic research on the long-term impact of early feeding modalities—particularly specific breastfeeding patterns—on infant GI health remains limited.

This study aimed to investigate the associations between feeding patterns at one month of age and gastrointestinal symptoms as well as overall burden during the first year of life. These findings may provide an evidence-based foundation for optimizing early infant feeding strategies, alleviating GI discomfort, and promoting healthy development in early life.

## 2. Materials and Methods

### 2.1. Study Design

This study was based on the Phoenix study (clinicaltrials.gov identifier: NCT05235412), which is a multicenter observational cohort conducted in six cities in China- Nanjing, Chengdu, Xi’an, Guangzhou, Wuhan, and Qingdao in order to explore human milk composition, health outcomes, and feeding practices of mothers and infants. A total of 779 mother–infant pairs were initially enrolled for long-term follow-up, of which 669 pairs were finally included in the present analysis. Details of the study design have been described previously [12].

The inclusion criteria for mothers were age ≥ 18 years, good general health, willingness to breastfeed, and provision of signed informed consent. Infants were eligible if they were born full-term (gestational age 37–42 weeks), healthy, breastfed at enrollment, and if both parents were of Chinese nationality. The exclusion criteria included participation in other interventional studies before enrollment; maternal severe medical conditions, breast disorders, psychiatric illness, or substance dependence; prepregnancy or early gestation (≤16 weeks) BMI < 18.5 or ≥28 kg/m^2^; nonsingleton pregnancy or assisted reproductive conception; and major congenital anomalies, chromosomal abnormalities, or exclusively formula-fed at birth.

The data used in this analysis were collected at four scheduled follow-up visits conducted at 1 month (±1 week), 4 months (±1 week), 6 months (±1 week), and 12 months (±2 weeks) of age (V1–V4). Additionally, a comprehensive baseline assessment of maternal and infant characteristics was performed at the time of enrollment.

### 2.2. Exposure Data Collection and Grouping

At the V1 visit, data on infant feeding practices during the first month of life were collected via maternal recall. These included whether the infant was exclusively breastfed, whether breast milk was fed via a bottle, and whether infant formula was introduced.

Feeding practices at 1 month of age were categorised into three groups as follows: Exclusive direct breastfeeding (EDB): Infants who were exclusively breastfed during the first month and received breast milk directly from the breast, without the use of bottles or other non-direct feeding methods. Bottle-fed expressed breastmilk (EBB): Infants who were exclusively breastfed, but received breast milk through a bottle, either solely by bottle or in combination with direct breastfeeding. Thus, the EBB group includes both exclusively bottle-fed infants and those fed by a mix of bottle and direct breastfeeding. To characterize within-group heterogeneity, breastfeeding and bottle-feeding frequencies (times/day) during the past week at the 1-month visit were collected (Supplementary Table S1). Mixed feeding (MF): Infants who received infant formula or other milk substitutes in addition to breast milk.

In the MF group, the specific types of infant formulas administered at the 1-month visit were documented. Breastfeeding frequency (times per day) was also collected; however, the precise volume of breast milk was not available. To explore potential heterogeneity within the MF group, Breastfeeding frequency was used as a proxy, and MF infants were stratified into breastmilk-dominant (>80% of feeds) and formula-dominant (≤80% of feeds) subgroups based on the median proportion at 1 month. These analyses are presented in the Supplementary Materials (Tables S2 and S3).

### 2.3. Collection and Definition of Outcome Data

Gastrointestinal (GI) symptoms were assessed using two validated instruments. The primary outcome was the Infant Gastrointestinal Symptom Questionnaire (IGSQ), which evaluates overall GI burden. The IGSQ consists of 13 items assessing GI distress over the past week, each scored from 1 to 5 (total range 13–65), with higher scores indicating greater burden [13]. Questionnaires with ≥10 completed items were included, and scores were normalized as: Total Score = (13/number of answered items) × sum of scores of answered items.

Secondary outcomes were six individual GI symptoms (bloating, swallowing difficulty, constipation, diarrhoea, and vomiting), assessed using relevant items extracted from the Pediatric Quality of Life Inventory™ (PedsQL™) Infant Scales [14]. These items capture whether the infant had experienced each symptom during the past month. According to the PedsQL™ developer guidelines, response options are: “never” (no occurrence), “rarely” (1–2 times per month), “sometimes” (several times but not weekly), “often” (several times per week), and “almost always” (daily or almost daily). In the main analysis, symptoms were dichotomized as absent (“never”) versus present (any of “rarely” to “almost always”).

### 2.4. Statistical Analysis

Missing data were handled using available-case analysis (i.e., all observed data at each time point were included without imputation), given that the proportion of missing data was low.

Continuous variables with normal distribution are presented as mean ± SD and compared by ANOVA; non-normally distributed variables as median (IQR) and compared by Kruskal–Wallis test. Categorical variables are expressed as *n* (%) and compared by the χ^2^ test. To quantify the magnitude of baseline differences among the three feeding groups, standardized mean differences (SMDs) were calculated for continuous and binary variables, and Cramér’s V was used for multinomial categorical variables. An SMD or Cramér’s V greater than 0.1 was considered indicative of meaningful imbalance. To assess potential attrition bias, baseline characteristics were compared between participants included in the final analysis and those excluded, using independent *t*-tests (for continuous variables) or χ^2^ tests (for categorical variables).

To estimate the effects of different feeding practices on infants’ IGSQ scores at 1, 4, 6, and 12 months of age, linear regression models were applied, with the results expressed as estimated β-effect values and 95% confidence intervals (95% CIs). Furthermore, to explore the associations between early feeding practices and the risk of developing specific gastrointestinal symptoms—including bloating, vomiting, constipation, diarrhoea, and swallowing difficulty—at each age point, binary logistic regression models were constructed, yielding odds ratios (ORs) and corresponding 95% CIs.

Considering the repeated measurements over time, generalized estimating equations (GEEs) with an exchangeable correlation structure were used to assess the longitudinal impact of feeding practices on IGSQ scores and individual symptoms from 1 to 12 months of age. The working correlation structure was selected based on the Quasi-likelihood under Independence Model Criterion (QIC); the exchangeable structure yielded the lowest QIC value (Supplementary Table S4) and was therefore used for all primary GEE analyses. To assess whether the effect of feeding mode varied with age, GEE models including a feeding mode × time interaction term were additionally tested.

To control for potential confounders, three progressively adjusted models were constructed: Model 1 was unadjusted; Model 2 was adjusted for the mother’s age (continuous) and education level (categorical); and Model 3 was fully adjusted on the basis of a minimally sufficient adjustment set identified through a directed acyclic graph (DAG), integrating prior literature and clinical expertise. The covariates included the mother’s age (continuous), education level (categorical), occupation (categorical), annual household income (categorical), gravidity (categorical), primiparity (categorical), delivery mode (categorical), presence of pregnancy complications (categorical), time to initiation of breastfeeding (continuous), and infant sex (categorical) (Supplementary Figure S1).

Several sensitivity analyses were performed. First, given that feeding practices may change during the first year of life, a time-varying exposure analysis was conducted in which infants were regrouped according to the feeding mode reported at each visit to assess the impact of dynamic changes in feeding practices on gastrointestinal outcomes. In this analysis, because no infant was exclusively formula-fed (FF) at 1 month of age and both FF and mixed feeding (MF) represent non-direct breastfeeding patterns, the FF and MF groups were combined into a single category (MF/FF). Second, to explore the potential impact of reverse causation, we excluded the 1-month outcome data and additionally adjusted for the corresponding symptom at 1 month (or baseline IGSQ score) in longitudinal GEE models for outcomes at 4, 6, and 12 months. Third, to control for potential confounding by probiotic use, time-varying probiotic use (yes/no at each visit) was additionally adjusted for in the main GEE models. Fourth, to test the robustness of the symptom dichotomization threshold, a stricter symptom definition was applied: a symptom was considered “present” only when its frequency was reported as “sometimes”, “often”, or “almost always”; the response “rarely” (1–2 times per month) was reclassified as “absent”. The results of this stricter definition were compared with those of the original analysis (where “rarely” was considered present). The results of these sensitivity analyses are presented in the Supplementary Materials.

All statistical analyses were performed using SPSS Statistics 26.0.1.0 (IBM Corp., Armonk, NY, USA). A two-sided *p* < 0.05 was considered statistically significant.

### 2.5. Quality Control

Researchers collected data via structured questionnaires. All questionnaires were completed by the infant‘s mother in person at each visit (the study protocol required her attendance for biological sample collection and explicitly prohibited other caregivers from completing the questionnaires on her behalf). Interviewers were not blinded to the infants’ feeding group, because they recorded detailed feeding behaviors (direct breastfeeding, bottle-feeding of expressed breast milk, or formula introduction) using the structured questionnaires at each visit. To minimize potential information bias, all team members completed standardized training before commencing their tasks, mastered the data collection procedures, and used closed-ended questions without probing or interpretation during the visits. During each visit, trained researchers provided face-to-face guidance and reviewed each questionnaire item to ensure data completeness and accuracy. After completion, both the researcher and the participant (the mother) signed the form. All visits followed the procedures outlined in the study manual. For visits that exceeded the scheduled window, included missing samples, or had incomplete questionnaires, the research team recorded the visit ID, date, and reason. In cases of early participant withdrawal, they documented the time and reason for withdrawal. The data collected before withdrawal remained valid, but the study team did not collect any additional information thereafter.

Researchers entered all questionnaire data into the electronic data capture (EDC) system, with each account uniquely linked to an individual researcher to ensure traceability. The Clinical Research Associate (CRA) performed source data verification (SDV) on 100% of the data submitted by each research center. To maintain protocol compliance and ensure data reliability, the project team also conducted routine quality checks, joint monitoring visits, and formal audits.

### 2.6. Ethics Approval and Standard Protocol Approval

The Phoenix study was conducted in accordance with the principles of the Declaration of Helsinki and received approval from the ethics committees of all the institutions hosting the participating centres. Informed consent was obtained from all the participating mothers, who provided consent and acted on behalf of their infants. The study procedures and reporting strictly adhered to the Strengthening the Reporting of Observational Studies in Epidemiology (STROBE) guidelines [15].

## 3. Results

### 3.1. Baseline Characteristics of the Study Population

**Figure 1:**
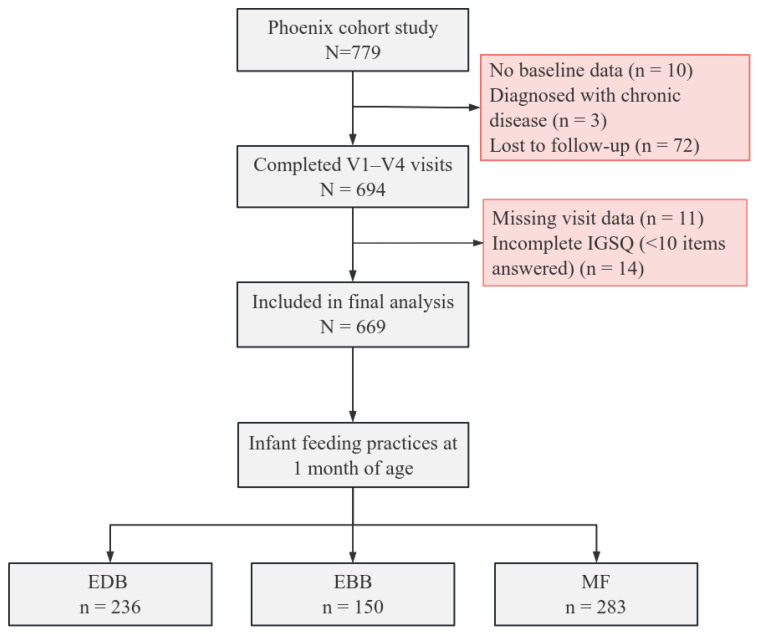
Subject selection flowchart (http://baidu.zaabj.com/, accessed on 17 April 2026). The gray boxes indicate participants included in the final analysis, whereas the red boxes indicate those excluded. EDB = exclusive direct breastfeeding; EBB = bottle-fed expressed breastmilk; MF: mixed feeding.

The subjects were from the Phoenix cohort (*n* = 779). Of these, 10 enrolled pairs had no available baseline data and were excluded from all analyses. Based on the inclusion and exclusion criteria, 3 pairs were excluded because of chronic prepregnancy conditions, and another 72 pairs did not complete the follow-up. Another 11 pairs with missing visit data and 14 pairs who answered fewer than 10 items on the IGSQ were also excluded. Finally, 669 mother–infant pairs were included for analysis. Participants were classified into three groups according to the infant’s feeding method at 1 month of age: 236 pairs (35.28%) in the EDB group, 150 pairs (22.42%) in the EBB group, and 283 pairs (42.30%) in the MF group (Figure 1).

Baseline characteristics were compared between the 669 included participants and the 100 excluded participants who had available baseline data (10 enrolled pairs had no baseline data and were excluded from this comparison). No significant differences were observed between the two groups in terms of feeding group distribution, maternal age, education, income, gravidity, parity, pregnancy complications, infant sex, delivery mode, or time to breastfeeding initiation (all *p* > 0.05; Supplementary Table S5).

Baseline characteristics were compared across the three groups. The mean maternal age was 30.63 ± 3.74 years. Most mothers held an undergraduate degree (44.69%) and were employed (73.54%). Approximately half of the households (50.07%) reported an annual income between 150,000 and 290,000 CNY. Primiparous mothers accounted for 60.84% of the sample. The rate of vaginal delivery (55.61%) was slightly higher than that of caesarean section (44.39%). A total of 32.14% of mothers reported pregnancy complications. The median time to initiation of breastfeeding was 1.00 (IQR: 1.00, 3.00) days postpartum. With respect to infant characteristics, sex was nearly evenly distributed, with 51.87% being male and 48.13% being female. The mean birth weight was 3342.25 ± 369.80 g, and the mean birth length was 50.00 ± 1.22 cm. Additionally, 6.13% (*n* = 41) of the infants were diagnosed with common neonatal diseases. Statistically significant differences were observed among the feeding groups concerning annual household income, gravidity, primiparous, mode of delivery, and time to initiation of breastfeeding (*p* < 0.05) (Table 1).

Variables with |SMD| > 0.1 included time to breastfeeding initiation, gravidity, parity, annual household income, maternal age, pregnancy complications, delivery mode, neonatal diseases, and infant sex (Supplementary Table S6).

### 3.2. The Comparison of Gastrointestinal Symptom Burden Among Three Groups

**Figure 2:**
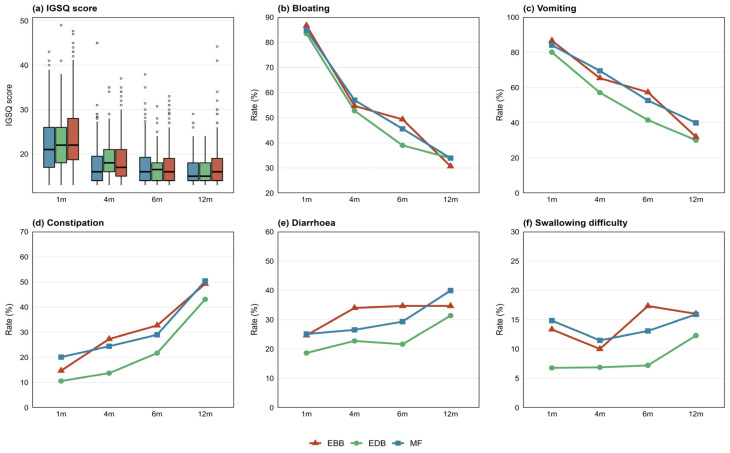
Changes in infant gastrointestinal outcomes across different follow-up times. (**a**) IGSQ scores (presented as box plots: boxes = interquartile range [P25–P75], horizontal lines = medians, whiskers = most extreme non-outlier points). (**b**) Rate of bloating. (**c**) Rate of vomiting. (**d**) Rate of constipation. (**e**) Rate of diarrhoea. (**f**) Rate of swallowing difficulty. For panels (**b**)–(**f**): Data are shown as point-line graphs (points = observed rate at each time point; lines = trend over time). EDB, exclusive direct breastfeeding; EBB, exclusive breastfeeding with bottle; MF, mixed feeding.

The gastrointestinal burden among infants, as reflected by IGSQ scores, progressively decreased with age across all the feeding groups. However, throughout the follow-up period, infants in the EDB group consistently presented the lowest IGSQ scores, suggesting better gastrointestinal adaptation. Consistent with the IGSQ trends, the incidence of bloating and vomiting generally decreased over time, whereas the occurrence of constipation, diarrhoea, and swallowing difficulty increased with age. Notably, the EDB group maintained a relatively favourable profile across all symptoms, with the lowest rates observed throughout the study period. Before 6 months of age, infants in the EBB group presented lower IGSQ scores and a lower incidence of bloating, vomiting, and swallowing difficulty than did those in the MF group. After 6 months, however, this trend reversed, with the EBB group showing higher IGSQ scores and more frequent symptoms than the MF group did (Figure 2). The absolute frequencies (*n*/N, %) of each symptom by feeding group and age are provided in Supplementary Table S7.

### 3.3. Age-Specific Associations Between Feeding Practices and IGSQ Scores: Stepwise Adjusted Linear Regression Analysis

To further investigate the associations between early feeding practices and infant gastrointestinal function, a multi-time-point analysis of IGSQ scores was conducted via linear regression models. The results are presented in Table 2.

At 1 month of age, the MF group was associated with significantly higher IGSQ scores compared with the EDB group across all three models (Model 3: β = 0.668, 95% CI: 0.093–1.243, *p* = 0.023). No significant difference was observed between the EBB and EDB groups.

At 4 months of age, a significant difference in IGSQ scores was observed only in Model 2 for the MF group (β = 0.453, 95% CI: 0.038–0.867, *p* = 0.033), whereas the EBB group was not significantly different from the EDB group (*p* > 0.050).

At 6 months of age, both the EBB and MF groups presented significantly higher IGSQ scores than did the EDB group across all the models.

At 12 months of age, no statistically significant associations were observed between feeding practices and IGSQ scores (*p* > 0.05)

### 3.4. Longitudinal Associations Between Feeding Practices and IGSQ Scores Until 12 Months of Age

According to the results of the GEE analysis based on follow-up data (Table 3), infants in the MF group had significantly higher IGSQ scores than those in the EDB group across all three models. In the fully adjusted model (Model 3), MF showed a β of 0.950 (95% CI: 0.356–1.543, *p* = 0.002). For the EBB group, elevated IGSQ scores were statistically significant only in the unadjusted model (Model 1: β = 0.726, 95% CI: 0.095–1.357, *p* = 0.024); after adjustment for covariates, the differences were attenuated and no longer significant (Model 3: β = 0.519, 95% CI: −0.114 to 1.153, *p* = 0.108). A GEE model including a feeding mode × time interaction term was tested. The interaction was not statistically significant (χ^2^ = 8.00, *p* = 0.247).

### 3.5. Age-Specific Associations Between Feeding Practices and Gastrointestinal Symptom Risks: Stepwise Adjusted Logistic Regression Analysis

**Figure 3:**
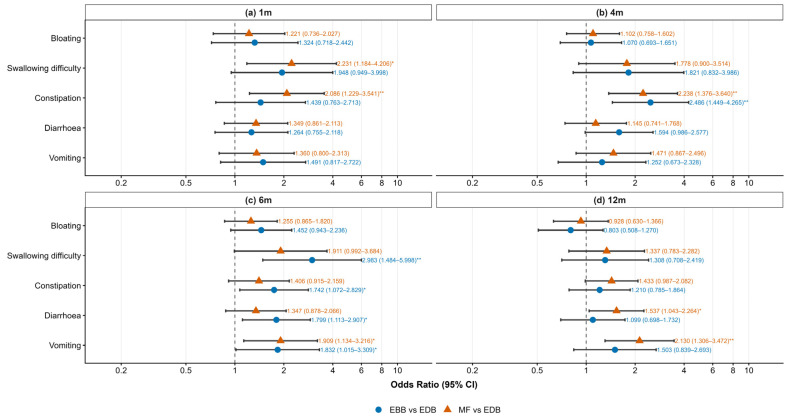
Associations between infant feeding practices and gastrointestinal symptoms at 1, 4, 6 and 12 months. Adjusted odds ratios (ORs) and 95% confidence intervals (CIs) for exclusive breastfeeding with bottle (EBB) and mixed feeding (MF) versus exclusive direct breastfeeding (EDB) are shown, faceted by time point: (**a**) 1 month, (**b**) 4 months, (**c**) 6 months, (**d**) 12 months. Within each panel, symptoms are ordered from top to bottom: Bloating, Swallowing difficulty, Constipation, Diarrhoea, Vomiting. Models adjusted for maternal age, education, occupation, income, gravidity, parity, mode of delivery, pregnancy complications, time to breastfeeding initiation, and infant sex. Dashed vertical line: OR = 1. Error bars: 95% CI. Blue circles: EBB vs. EDB; orange triangles: MF vs. EDB. OR and 95% CI values are displayed to the right of each point. * *p* < 0.05, ** *p* < 0.01.

To further investigate the associations between early feeding practices and gastrointestinal symptoms in infants at different ages, logistic regression models were employed to assess differences in symptom incidence across feeding groups. As shown in Figure 3, at 1 month of age, infants in the MF group had significantly higher odds of constipation than did those in the EDB group (OR = 2.09, 95% CI: 1.23–3.54; *p* = 0.006). Although the EBB group showed a trend toward higher odds of constipation, the difference was not statistically significant (OR = 1.44, 95% CI: 0.76–2.71; *p* = 0.260). Regarding swallowing difficulty, the MF group had significantly higher odds than the EDB group (OR = 2.23, 95% CI: 1.18–4.21, *p* = 0.013); the EBB group showed a trend toward higher odds that did not reach statistical significance (OR = 1.95, 95% CI: 0.95–4.00, *p* = 0.069). In contrast, no significant differences were observed among the feeding groups for bloating, vomiting, or diarrhoea (*p* > 0.050).

At 4 months of age, both the EBB and MF group had significantly higher odds of constipation than the EDB group (EBB: OR = 2.49, 95% CI: 1.45–4.27, *p* = 0.001; MF: OR = 2.24, 95% CI: 1.38–3.64, *p* = 0.001). For swallowing difficulty, the odds were higher in the MF group but did not reach statistical significance (OR = 1.78, 95% CI: 0.90–3.51, *p* = 0.098). Consistently, no statistically significant differences were observed among the groups in terms of bloating, vomiting, or diarrhoea (*p* > 0.05).

At 6 months of age, infants in the EBB group had significantly higher odds of swallowing difficulty (OR = 2.98, 95% CI: 1.48–6.00, *p* = 0.002), constipation (OR = 1.74, 95% CI: 1.07–2.83, *p* = 0.025), diarrhoea (OR = 1.80, 95% CI: 1.11–2.91, *p* = 0.017), and vomiting (OR = 1.83, 95% CI: 1.01–3.31, *p* = 0.045). In the MF group, significantly higher odds were observed for vomiting (OR = 1.91, 95% CI: 1.13–3.22, *p* = 0.015), while the odds for constipation (OR = 1.41, 95% CI: 0.92–2.16, *p* = 0.120) and diarrhoea (OR = 1.35, 95% CI: 0.88–2.07, *p* = 0.173) did not reach statistical significance.

At 12 months of age, no significant increase in gastrointestinal symptom odds was observed in the EBB group (all *p* > 0.05). However, infants in the MF group had significantly higher odds of vomiting (OR = 2.13, 95% CI: 1.31–3.47, *p* = 0.002) and diarrhoea (OR = 1.54, 95% CI: 1.04–2.26, *p* = 0.030).

Longitudinal analysis via logistic GEE across the entire follow-up period revealed that infants in the EBB and MF groups had higher odds of vomiting, constipation, diarrhoea, and swallowing difficulty compared with those in the EDB group (Supplementary Figure S2).

### 3.6. Sensitivity Analyses

Adjustment for baseline symptoms. To assess the potential impact of reverse causation, outcome data at 1 month of age were excluded, only outcomes at 4, 6, and 12 months were analyzed, and the corresponding symptom at 1 month (or baseline IGSQ score) was adjusted for as a baseline covariate in longitudinal GEE models. After adjustment, the associations of EBB with constipation, diarrhoea, and vomiting were no longer significant, whereas MF remained significantly associated with constipation, vomiting, swallowing difficulty, and IGSQ score. Notably, bloating became significant for both EBB (OR = 1.37, 95% CI: 1.04–1.82, *p* = 0.028) and MF (OR = 1.31, 95% CI: 1.06–1.62, *p* = 0.014) after adjustment. Detailed results are presented in Supplementary Table S8a,b.

Time-varying exposure analysis. The distribution of feeding practices at 1, 4, 6, and 12 months of age is shown in Supplementary Table S9a. To assess whether changes in feeding practices after 1 month influenced the findings, a time-varying exposure analysis was conducted using feeding mode updated at each visit. As shown in Supplementary Table S9b, the association for IGSQ score remained directionally consistent with the main analysis, with a reduced effect size (e.g., MF vs. EDB: β changed from 0.950 to 0.679). For gastrointestinal symptoms (Table 4), the time-varying analysis revealed that EBB was no longer significantly different from EDB for vomiting, constipation, or diarrhoea. In contrast, bloating remained significantly higher in the EBB group (OR = 1.31, *p* = 0.042) and swallowing difficulty showed a borderline trend (OR = 1.48, *p* = 0.056). For the MF group, differences from EDB persisted for most symptoms, albeit with attenuated effect sizes.

Adjustment for probiotic use. Probiotic use across feeding groups and ages is shown in Supplementary Table S10a. To evaluate whether probiotic use confounded the associations, time-varying probiotic use (yes/no at each visit) was additionally adjusted for in the main GEE models (Supplementary Table S10b,c). After adjustment, the effect estimates for feeding practices remained virtually unchanged (e.g., MF vs. EDB on IGSQ: β changed from 0.950 to 0.943; vomiting OR from 1.70 to 1.68; all changes < 5%), and all statistical conclusions were consistent.

Stricter symptom definition. To assess the robustness of the symptom dichotomization, symptoms were re-defined as present only when reported as “sometimes,” “often,” or “almost always” (excluding “rarely”). Longitudinal GEE analyses under this stricter definition are presented in Supplementary Figure S2. The association between mixed feeding and constipation remained statistically significant (OR = 1.40, 95% CI: 1.01–1.93, *p* = 0.041). For most other symptoms, the odds ratios were attenuated and no longer significant. The association for swallowing difficulty reversed direction (OR < 1) under the stricter definition.

## 4. Discussion

Early infant feeding practices were associated with gastrointestinal symptoms and overall burden during the first 12 months of life. The EDB group consistently exhibited the lowest burden of gastrointestinal symptoms and emerged as the optimal mode. The MF group was robustly associated with higher IGSQ scores and increased odds of constipation, vomiting, and swallowing difficulty. The EBB group had only a suggestive association with bloating; its associations with other symptoms were likely influenced by reverse causation or exposure misclassification. These findings indicate that EDB is the most protective mode for infant gastrointestinal health. MF was robustly associated with increased odds of multiple gastrointestinal symptoms, whereas the impact of EBB was limited, mainly confined to bloating.

Infants in the EDB group consistently had lower IGSQ scores and lower odds of most gastrointestinal symptoms across different ages, particularly during the first six months of life. Longitudinal analyses showed that IGSQ scores in the EDB group remained lower than those in the MF group throughout the follow-up period. Although some differences did not reach statistical significance at individual time points, the overall trend was evident, suggesting that EDB was associated with lower gastrointestinal burden in infants. This finding aligns with the results of a multinational, multicenter study [16], which also reported lower IGSQ scores in breastfed infants than in formula-fed infants (without distinguishing between direct and bottle-feeding). However, our study further shows that even formula feeding (MF group) is associated with a higher gastrointestinal burden, and that direct breastfeeding (EDB) confers a more pronounced protective effect.

In terms of specific symptoms, the EDB group showed favourable associations with constipation and swallowing difficulty. The odds of constipation in the MF group were more than twice those in the EDB group at 1 and 4 months of age, a disparity that highlights the favourable association with EDB. This aligns with existing evidence identifying breastfeeding as a factor associated with lower constipation incidence during infancy [17,18,19]. This disparity may be attributed to differences in the fat composition of formula milk and the absence of bioactive components found in breast milk [20]. Mechanistically, breast milk contains a wide range of bioactive components that are beneficial to intestinal health. For example, sn-2 position esterified palmitic acid facilitates fat absorption and promotes intestinal peristalsis [21]; human milk oligosaccharides (HMOs) support the colonization of beneficial bacteria while inhibiting the growth of pathogenic species [22]; and immune factors such as lactoferrin and secretory immunoglobulin A (sIgA) help to strengthen intestinal barrier function [23,24,25,26]. In addition, studies have demonstrated that the gut microbiota of exclusively breastfed infants is predominantly composed of bifidobacteria, which contribute to microbial homeostasis and reduce inflammation [27]. These mechanisms may collectively create a favourable digestive environment, which is particularly critical during early infancy when gastrointestinal function remains immature [28].

However, the situation becomes more complex when breast milk is delivered via a bottle. In the present study, the EBB group exhibited elevated rates of several gastrointestinal symptoms after 6 months of age, and in the main analysis, these rates even exceeded those of the MF group (Figure 2). This crossover phenomenon requires cautious interpretation. Six months is a period of complementary food introduction, and bottle-fed infants may have poorer oral suction capacity than directly breastfed infants [29]. Moreover, conventional bottles may disrupt the natural coordination of sucking, swallowing, and breathing, increasing the risk of air swallowing during feeding [30]. However, these physiological factors alone do not fully explain the reversal. More importantly, feeding practices may change over time (exposure misclassification), and early-onset symptoms may influence feeding choices (reverse causation), potentially biasing the true associations in the main analysis [31]. Sensitivity analyses revealed that the associations of EBB with vomiting, constipation, and diarrhoea were largely non-significant, suggesting that the elevated rates of these symptoms in the main analysis may be primarily driven by exposure misclassification or reverse causation rather than an independent effect of bottle-feeding per se. In contrast, bloating remained significant in multiple sensitivity analyses (time-varying: OR = 1.31; adjustment for baseline symptoms: OR = 1.37), representing the only relatively robust association for EBB. Under the stricter symptom definition, the odds ratio for swallowing difficulty reversed direction (OR < 1). This is likely due to the substantially reduced number of positive events under the stricter definition, leading to unstable estimates; this finding does not negate the clinical relevance of the mild symptom differences observed in the main analysis. In clinical and epidemiological surveys, caregivers typically report infant symptoms based on whether they occurred rather than on precise frequency [32]. Therefore, the main definition, which classifies “rarely” (1–2 times per month) as “present”, better reflects real-world reporting practices, and these mild symptoms may still affect infant comfort and caregiver burden.

Time-varying exposure analysis, which adjusts for changes in feeding practices over time, may provide estimates closer to the independent effect of bottle-feeding behavior. This analysis showed that the association between EBB and bloating became somewhat stronger (OR = 1.31), while swallowing difficulty showed a borderline trend (OR = 1.48, *p* = 0.056), while the associations with vomiting, constipation, and diarrhoea were no longer significant. This pattern has biological plausibility: the faster flow rate during bottle-feeding makes it difficult for infants to regulate their sucking rhythm, leading to increased air swallowing (aerophagia) and potentially causing bloating [33]; moreover, fewer oral muscles are involved in bottle-feeding than in direct breastfeeding, which may challenge the infant’s oral motor coordination and result in perceived swallowing difficulty [29]. Furthermore, bottle-fed infants require more frequent sucks to rebuild oral vacuum, further increasing the risk of aerophagia [34]. In contrast, the associations of EBB with vomiting, constipation, and diarrhoea were no longer significant in sensitivity analyses, suggesting that the elevated rates of these symptoms in the main analysis may be primarily attributable to exposure misclassification or reverse causation rather than an independent effect of bottle-feeding per se. Therefore, it is speculated that direct breastfeeding may have a protective effect against bloating (and, to some extent, swallowing difficulty), while bottle-feeding of expressed breastmilk may slightly increase the risk of these symptoms (bloating OR = 1.31). In clinical practice, for mothers who cannot directly breastfeed and choose to bottle-feed expressed breastmilk, they may be informed of a possible slight increase in bloating risk but need not be overly concerned; simple measures such as paced feeding and adequate burping may help reduce discomfort [33]. It should be noted, however, that the evidence for swallowing difficulty is limited (borderline significance), and its clinical significance warrants further validation.

Based on the above findings, this study emphasizes that the EDB is the optimal strategy for protecting infant gastrointestinal health and should be prioritized. For situations where bottle-feeding is unavoidable, caregivers should be provided with evidence-based guidance through health education and professional support, including paced feeding, selection of nipples with appropriate flow rates, and recognition of satiety cues [34,35]. These practices may help optimize bottle-feeding behavior and reduce gastrointestinal discomfort.

This study has several notable strengths. First, it systematically tracked the occurrence of gastrointestinal symptoms in infants throughout their first 12 months of life, integrating both cross-sectional and longitudinal analyses to dynamically evaluate the impact of different feeding practices on gastrointestinal health. Second, the study employed a refined classification of feeding practices—exclusive direct breastfeeding, exclusive bottle feeding, and mixed feeding—which goes beyond the traditional “breastmilk versus formula” dichotomy, and provides evidence for an independent association of feeding mode and gastrointestinal tolerance. Additionally, the IGSQ was used to assess overall gastrointestinal function alongside specific symptoms, yielding a more comprehensive picture of the associations.

Several limitations should be acknowledged. First, reporting bias and blinding. Gastrointestinal symptoms relied on caregiver reports, which may introduce recall bias. Moreover, interviewers were not blinded to feeding groups, which may have introduced information bias, particularly for subjective symptom assessment. Second, residual confounding. Unmeasured factors such as maternal return-to-work timing, bottle nipple flow rate, infant latch difficulties, and whether the formula contained prebiotics or probiotics may influence both feeding mode and outcomes. Third, reverse causation and exposure misclassification. Although a sensitivity analysis excluding 1-month outcomes and adjusting for baseline symptoms was conducted to test reverse causation, the possibility of residual reverse causation cannot be completely ruled out. Furthermore, feeding practices were assessed only at fixed visit time points, and the exact timing of any switches between visits was not recorded; therefore, some degree of exposure misclassification cannot be entirely excluded. Fourth, limited subgroup sample sizes or low symptom incidence. For example, the subgroups within the MF group stratified by breastfeeding frequency had relatively small sample sizes, which may affect the stability of estimates. Moreover, the low incidence of swallowing difficulty led to a substantial reduction in positive events under the stricter symptom definition, resulting in unstable estimates. Fifth, heterogeneity of the EBB group and missing information. The EBB group was heterogeneous in composition, which may have attenuated effect estimates. Moreover, the specific reasons for choosing bottle-feeding were not recorded.

## 5. Conclusions

In this prospective cohort of 669 infants, exclusive direct breastfeeding (EDB) was associated with the lowest gastrointestinal symptom burden throughout the first year. Mixed feeding (MF) was robustly associated with higher IGSQ scores and increased risks of constipation, vomiting, and swallowing difficulty. For bottle-fed expressed breastmilk (EBB), only bloating remained significantly elevated after sensitivity analyses (OR = 1.31). Direct breastfeeding is the optimal mode for infant gastrointestinal health. When bottle-feeding is unavoidable, paced feeding and adequate burping may help reduce discomfort.

## Figures and Tables

**Table 1:** Baseline characteristics of the participants.

Characteristics	Total Population *n* = 669	EDB *n* = 236	EBB *n* = 150	MF *n* = 283	*p*
Mothers					
Age	30.63 ± 3.74	30.52 ± 3.78	30.12 ± 3.29	30.99 ± 3.92	0.061
Education level					0.071
High school or below	84 (12.56)	37 (15.68)	13 (8.67)	34 (12.01)	
Junior college	170 (25.41)	61 (25.85)	42 (28.00)	67 (23.67)	
Undergraduate	299 (44.69)	107 (45.34)	69 (46.00)	123 (43.46)	
Postgraduate or above	116 (17.34)	31 (13.14)	26 (17.33)	59 (20.85)	
Occupation					0.450
Employed	492 (73.54)	170 (72.03)	109 (72.67)	213 (75.27)	
Self-employed	46 (6.88)	16 (6.78)	10 (6.67)	20 (7.07)	
Housewife	100 (14.95)	43 (18.22)	21 (14.00)	36 (12.72)	
Other	31 (4.63)	7 (2.97)	10 (6.67)	14 (4.95)	
Annual household income					0.005
<150,000	222 (33.18)	94 (39.83)	41 (27.33)	87 (30.74)	
150,000–299,999	335 (50.07)	114 (48.31)	78 (52.00)	142 (50.53)	
≥300,000	112 (16.74)	28 (11.86)	31 (20.67)	53 (18.73)	
Gravidity					<0.001
<2 pregnancies	528 (78.92)	168 (71.19)	131 (87.33)	229 (80.92)	
≥2 pregnancies	141 (21.08)	68 (28.81)	19 (12.67)	54 (19.08)	
Primiparous					<0.001
Yes	407 (60.84)	119 (50.42)	104 (69.33)	184 (65.02)	
No	262 (39.16)	117 (49.58)	46 (30.67)	99 (34.98)	
Delivery mode					0.192
Vaginal delivery	372 (55.61)	142 (60.17)	82 (54.67)	148 (52.30)	
Cesarean section	297 (44.39)	94 (39.83)	68 (45.33)	135 (47.70)	
^a^ Pregnancy complications					0.091
Yes	215 (32.14)	68 (28.81)	43 (28.67)	104 (36.75)	
No	454 (67.86)	168 (71.19)	107 (71.33)	179 (63.25)	
Time to initiation of breastfeeding	1.00 (1.00, 3.00)	1.00 (1.00, 2.00)	1.00 (1.00, 2.00)	2.00 (1.00, 3.00)	<0.001
Infants					
Sex					0.589
Male	347 (51.87)	118 (50.00)	83 (55.33)	146 (51.59)	
Female	322 (48.13)	118 (50.00)	67 (44.67)	137 (48.41)	
Birth weight (g)	3342.25 ± 369.80	3348.17 ± 367.35	3348.32 ± 392.40	3334.14 ± 360.61	0.914
Birth length (cm)	50.00 ± 1.22	50.00 ± 1.09	49.99 ± 1.36	49.92 ± 1.24	0.772
^b^ neonatal diseases					0.208
Yes	41 (6.13)	17 (7.20)	12 (8.00)	12 (4.24)	
No	628 (93.87)	219 (92.80)	138 (92.00)	271 (95.76)	

Categorical variables are expressed as *n* (%). Continuous variables are presented as the mean ± standard deviation (SD), if normally distributed or as the median and interquartile range (IQR) if nonnormally distributed. Group comparisons were conducted via one-way ANOVA for normally distributed continuous variables, the Kruskal–Wallis test for nonnormally distributed variables, and the chi-square (χ^2^) test for categorical variables. *p* values were derived from comparisons among the three feeding groups (EDB, EBB, and MF). A two-sided *p* value < 0.05 was considered statistically significant. ^a^ Pregnancy complications include gestational diabetes mellitus, gestational hypertension, gestational hypothyroidism, preeclampsia/eclampsia, and other related conditions. ^b^ Neonatal diseases include physician-diagnosed conditions occurring within the first 28 days of life, such as neonatal jaundice, neonatal pneumonia, infections, and mild asphyxia.

**Table 2:** Associations between IGSQ scores and feeding practices at 1, 4, 6, and 12 months based on stepwise adjusted linear regression models.

Feeding Practices	Model 1		Model 2		Model 3	
	β (95% CI)	*p*	β (95% CI)	*p*	β (95% CI)	*p*
1 month						
EDB	Ref.	-	Ref.	-	Ref.	-
EBB	1.058 (−0.283, 2.399)	0.122	0.810 (−0.505, 2.125)	0.227	0.599 (−0.748, 1.946)	0.383
MF	0.753 (0.187, 1.319)	0.009	0.784 (0.228, 1.340)	0.006	0.668 (0.093, 1.243)	0.023
4 month						
EDB	Ref.	-	Ref.	-	Ref.	-
EBB	0.504 (−0.481, 1.488)	0.316	0.444 (−0.537, 1.425)	0.375	0.382 (−0.625, 1.388)	0.457
MF	0.413 (−0.003, 0.828)	0.052	0.453 (0.038, 0.867)	0.033	0.411 (−0.019, 0.841)	0.061
6 month						
EDB	Ref.	-	Ref.	-	Ref.	-
EBB	1.097 (0.295, 1.899)	0.007	1.049 (0.249, 1.849)	0.010	0.926 (0.106, 1.746)	0.027
MF	0.617 (0.278, 0.955)	<0.001	0.648 (0.309, 0.986)	<0.001	0.581 (0.231, 0.932)	0.001
12 month						
EDB	Ref.	-	Ref.	-	Ref.	-
EBB	0.245 (−0.541, 1.031)	0.541	0.176 (−0.603, 0.956)	0.657	0.164 (−0.636, 0.963)	0.688
MF	0.196 (−0.135, 0.528)	0.246	0.234 (−0.096, 0.563)	0.164	0.242 (0.099, 0.583)	0.164

Model 1: Unadjusted (crude model) Model 2: Adjusted for maternal age and education level Model 3: Further adjusted for maternal age, education level, occupation, annual household income, gravidity, primiparity, mode of delivery, pregnancy complications, time to initiation of breastfeeding, and infant sex. Ref: Reference group 95% CI: 95% confidence interval.

**Table 3:** Effect estimates for IGSQ scores by feeding practices on the basis of stepwise adjusted linear GEE models during follow-up (until 12 months of age).

Feeding Practices	Model 1		Model 2		Model 3	
	β (95% CI)	p	β (95% CI)	p	β (95% CI)	*p*
EDB	Ref.	-	Ref.	-	Ref.	-
EBB	0.726 (0.095, 1.357)	0.024	0.621 (0.001, 1.240)	0.050	0.519 (−0.114, 1.153)	0.108
MF	0.993 (0.415, 1.571)	0.001	1.058 (0.489, 1.628)	<0.001	0.950 (0.356, 1.543)	0.002

Model 1: Unadjusted (crude model). Model 2: Adjusted for maternal age and education level. Model 3: Further adjusted for maternal age, education level, occupation, annual household income, gravidity, primiparity, mode of delivery, pregnancy complications, time to initiation of breastfeeding, and infant sex. Ref: Reference group. 95% CI: 95% confidence interval.

**Table 4:** Sensitivity analysis using time-varying feeding exposure: comparison with main analysis (longitudinal GEE) for gastrointestinal symptoms.

		Main Analysis		Time-Varying Exposure Analysis	
Symptom	Comparison	OR (95% CI)	*p*	OR (95% CI)	*p*
Bloating	EBB vs. EDB	1.12 (0.84, 1.49)	0.438	1.31 (1.01, 1.71)	0.042
	MF vs. EDB	1.13 (0.84, 1.45)	0.341	1.28 (1.05, 1.57)	0.017
Vomiting	EBB vs. EDB	1.51 (1.04, 2.19)	0.032	1.14 (0.82, 1.58)	0.430
	MF vs. EDB	1.70 (1.26, 2.28)	<0.001	1.35 (1.05, 1.74)	0.020
Constipation	EBB vs. EDB	1.58 (1.17, 2.14)	0.003	1.09 (0.81, 1.47)	0.575
	MF vs. EDB	1.64 (1.27, 2.11)	<0.001	1.56 (1.23, 1.97)	<0.001
Diarrhoea	EBB vs. EDB	1.38 (1.05, 1.81)	0.020	1.21 (0.93, 1.59)	0.162
	MF vs. EDB	1.31 (1.02, 1.67)	0.032	1.22 (0.98, 1.53)	0.078
Swallowing difficulty	EBB vs. EDB	1.79 (1.24, 2.58)	0.004	1.48 (0.99, 2.22)	0.056
	MF vs. EDB	1.84 (1.22, 2.78)	0.002	1.77 (1.31, 2.39)	<0.001

Main analysis used feeding mode assessed at 1 month of age only (EDB, EBB, MF). Time-varying analysis used the actual feeding mode reported at 1, 4, 6, and 12 months. Exclusive FF was reclassified as MF Both models were adjusted for the same covariates as Model 3 (maternal age, education, occupation, income, gravidity, parity, delivery mode, pregnancy complications, time to breastfeeding initiation, infant sex) and used an exchangeable working correlation structure. The time-varying model did not include a feeding mode × time interaction term (*p* = 0.247); therefore, the reported effects represent average associations over the entire follow-up period. EDB = exclusive direct breastfeeding; EBB = bottle-fed expressed breastmilk; MF = mixed feeding; FF = exclusive formula feeding.

## Data Availability

The data presented in this study are available on request from the corresponding author. The data are not publicly available due to privacy and ethical restrictions.

## Supplementary Materials

The following supporting information can be downloaded at: https://www.mdpi.com/article/10.3390/nu18091383/s1, Table S1: Feeding characteristics of the EBB group at 1 month of age, Table S2: Association between breastfeeding frequency proportion and IGSQ scores within the mixed feeding group, Table S3: Association between breastfeeding frequency proportion and specific gastrointestinal symptoms at 1, 4, 6, and 12 months within the mixed feeding group, Table S4: Comparison of QIC values for different working correlation structures in GEE models for IGSQ scores, Table S5: Comparison of baseline characteristics between included and excluded participants, Table S6: Effect sizes for baseline comparisons among feeding groups (EDB, EBB, MF), Table S7: Absolute frequency of gastrointestinal symptoms stratified by feeding group (fixed at 1 month) and age, Table S8: (a) Longitudinal GEE sensitivity analysis: associations between feeding practices and gastrointestinal symptoms after adjusting for baseline symptoms (4–12 months) (b) Sensitivity analysis adjusting for baseline IGSQ score: comparison of main analysis and sensitivity analysis for IGSQ score, Table S9. (a) Feeding practices at 1, 4, 6, and 12 months of age. (b) Sensitivity analysis using time varying feeding exposure: comparison with main analysis (fixed 1 month exposure), Table S10. (a) Probiotic use by feeding group and age. (b) Sensitivity analysis adjusting for time‑varying probiotic use: comparison with main analysis for IGSQ score (linear GEE). (c) Sensitivity analysis adjusting for time‑varying probiotic use: comparison with main analysis for individual gastrointestinal symptoms (logistic GEE), Figure S1: Conceptual framework for covariate selection in the association between early feeding practices and infant gastrointestinal symptoms. Arrows indicate assumed causal pathways. Green arrows represent the direct causal effect of feeding practices (Exposure) on gastrointestinal symptoms (Outcome), which is the primary relationship of interest. Pink arrows denote potential confounding paths—i.e., shared antecedents of both the exposure and outcome—that should be blocked via statistical adjustment. Black arrows represent other causal pathways among covariates that do not constitute confounding. Based on the DAG structure and using DAGitty software, the minimally sufficient adjustment set identified includes:Maternal age, education level, occupation, annual household income, gravidity, primiparity, mode of delivery, pregnancy complications, time to initiation of breastfeeding, and infant sex DAG constructed using DAGitty v3.0 (http://www.dagitty.net/, accessed on 17 April 2026), Figure S2: Forest plot comparison of associations between feeding practices and gastrointestinal symptoms using original vs. strict definitions in longitudinal GEE analysis. (a) Original definition (symptoms include “rarely”). (b) Strict definition (only “sometimes”, “often”, or “almost always” considered as events). Adjusted odds ratios (ORs) and 95% confidence intervals (CIs) for EBB and MF versusEDB are shown. Models adjusted for maternal age, education, occupation, income, gravidity, parity, mode of delivery, pregnancy complications, time to breastfeeding initiation, and infant sex (exchangeable working correlation). Dashed vertical line: OR = 1. Error bars: 95% CI. Blue circles: EBB vs. EDB; orange triangles: MF vs. EDB. OR and 95% CI values are displayed to the right of each point. * *p* < 0.05, ** *p* < 0.01, *** *p* < 0.001.

## Abbreviations

EDB	Exclusive direct breastfeeding
EBB	Exclusive breastfeeding with bottle
MF	Mixed feeding
IGSQ	Infant Gastrointestinal Symptom Questionnaire
GEE	Generalized estimating equation
MDD	Minimum dietary diversity
DAG	Directed acyclic graph
FGIDs	Functional gastrointestinal disorders
