# Supplementary material for: Association of Early Feeding Practices with Gastrointestinal Symptoms in Infants During the First 12 Months: A Multicenter Prospective Cohort Study
Source: Nutrients. 2026 Apr 28;18(9):1383. doi: 10.3390/nu18091383 (PMC13164724; doi:10.3390/nu18091383)
Supplement: Supplementary file 1 [file nutrients-18-01383-s001.zip › nutrients-4206854-supplementary.pdf]

Table S1. Feeding characteristics of the EBB group at 1 month of age

Variable	Median (Q1, Q3)	Range
Breastfeeding frequency (times/day)	8.0 (5.0, 9.0)	0–15
Bottle-feeding frequency (times/day)	2.0 (1.0, 4.0)	0–5
Proportion of breastfeeding (%)	83.3 (60.0, 94.6)	0–100

Data are presented as median (interquartile range) and range. The EBB group includes infants who received exclusive breastmilk via bottle, encompassing both exclusively bottle-fed and mixed bottle-fed with direct breastfeeding. Feeding frequencies refer to the number of feeds per day during the past week at the 1-month visit. The proportion of breastfeeding was calculated as: [breastfeeding frequency / (breastfeeding frequency + bottle-feeding frequency)] × 100%. Sample size variation is due to missing breastfeeding frequency records for some infants. This table is intended solely to describe within-group heterogeneity and was not included in the main analyses.

Table S2. Association between breastfeeding frequency proportion and IGSQ scores within the mixed feeding group

Group	*n*	β	95% CI	*P*
Breastmilk-dominant (breastmilk frequency >80%)	131	Ref	-	-
Formula-dominant (breastmilk frequency ≤80%)	152	0.121	(-0.731, 0.973)	0.777

Adjusted for maternal age, education level, annual household income, gravidity, parity, pregnancy complications, infant sex, time to initiation of breastfeeding, and mode of delivery. The cut-off of 80% was based on the median proportion of breastmilk feeding frequency at the 1-month visit within the MF group. The outcome was IGSQ score at 1-12 month of age. This analysis demonstrates that within the MF group, the proportion of breastmilk vs. formula feeding frequency was not significantly associated with gastrointestinal burden.

Table S3. Association between breastfeeding frequency proportion and specific gastrointestinal symptoms at 1, 4, 6, and 12 months within the mixed feeding group

Symptom	Time point	OR (95% CI)	*P*
Bloating	1 month	0.88 (0.43–1.82)	0.731
	4 months	1.04 (0.61–1.76)	0.888
	6 months	0.60 (0.36–1.00)	**0.050**
	12 months	0.81 (0.47–1.39)	0.435
Vomiting	1 month	0.76 (0.38–1.52)	0.442
	4 months	0.50 (0.25–1.02)	0.057
	6 months	0.89 (0.48–1.66)	0.705
	12 months	0.81 (0.45–1.48)	0.498
Constipation	1 month	1.50 (0.79–2.83)	0.216
	4 months	0.59 (0.32–1.06)	0.079
	6 months	0.82 (0.48–1.42)	0.489
	12 months	1.35 (0.82–2.23)	0.244
Diarrhea	1 month	0.41 (0.23–0.76)	**0.004**
	4 months	0.63 (0.35–1.13)	0.122
	6 months	0.73 (0.42–1.27)	0.273
	12 months	1.16 (0.70–1.92)	0.564
Swallowing difficulty	1 month	1.17 (0.57–2.39)	0.674
	4 months	0.79 (0.35–1.82)	0.587
	6 months	0.67 (0.30–1.49)	0.327
	12 months	0.99 (0.48–2.04)	0.983

Reference group: Breastmilk-dominant (breastmilk feeding frequency >80% of total feeds at 1 month, *n*=131). Formula-dominant group defined as breastmilk feeding frequency ≤80% (*n*=152). All models adjusted for maternal age, education level, annual household income, gravidity, parity, pregnancy complications, infant sex, pet ownership, time to initiation of breastfeeding, and mode of delivery. Of the 20 comparisons, only diarrhea at 1 month reached statistical significance, and the direction (lower odds in formula-dominant group) was opposite to what would be expected if higher breastmilk proportion were protective. These findings collectively suggest that within the MF group, the proportion of breastmilk versus formula feeding is not a major determinant of gastrointestinal symptom risk.

Table S4. Comparison of QIC values for different working correlation structures in GEE models for IGSQ scores.

Working correlation structure	QIC
Exchangeable	61925
AR-1	61947
Unstructured	61929

The exchangeable structure yielded the lowest QIC and was therefore selected for all primary GEE analyses.

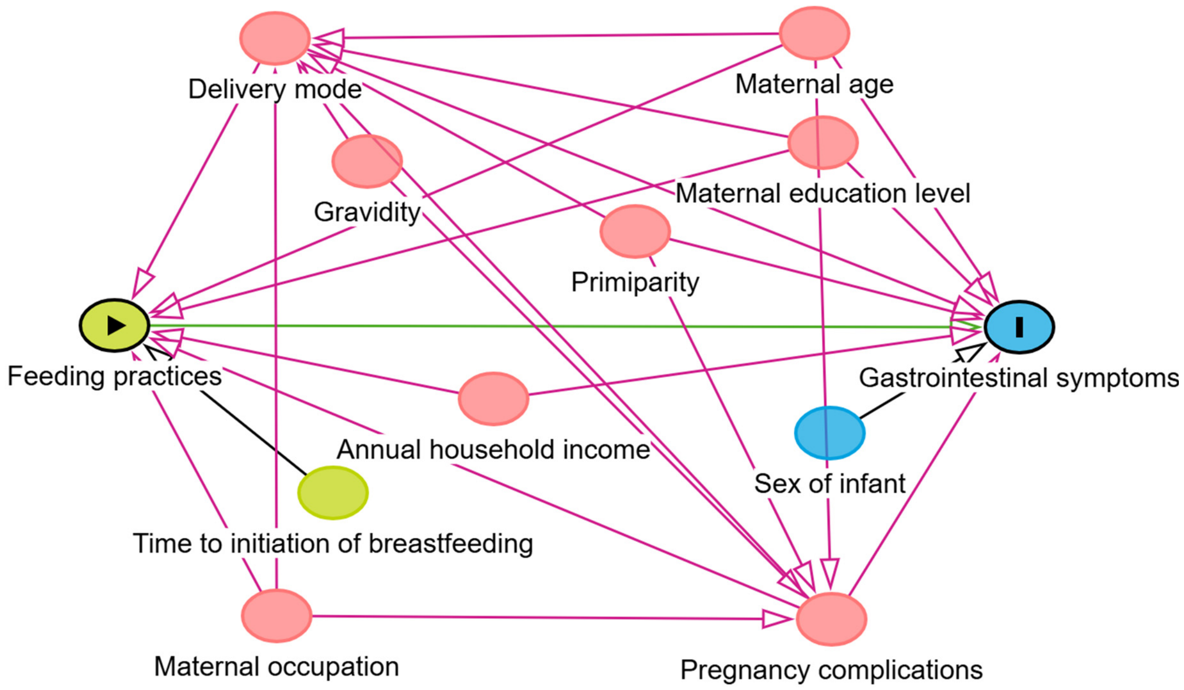

Figure S1. Conceptual framework for covariate selection in the association between early feeding practices and infant gastrointestinal symptoms

Arrows indicate assumed causal pathways.

Green arrows represent the direct causal effect of feeding practices (Exposure) on gastrointestinal symptoms (Outcome), which is the primary relationship of interest.

Pink arrows denote potential confounding paths—i.e., shared antecedents of both the exposure and outcome—that should be blocked via statistical adjustment.

Black arrows represent other causal pathways among covariates that do not constitute confounding.

Based on the DAG structure and using DAGitty software, the minimally sufficient adjustment set identified includes:Maternal age, education level, occupation, annual household income, gravidity, primiparity, mode of delivery, pregnancy complications, time to initiation of breastfeeding, and infant sex

DAG constructed using DAGitty v3.0 (http://www.dagitty.net/).

Table S5. Comparison of baseline characteristics between included and excluded participants

Characteristic	Included (*n*=669)	Excluded (*n*=100)	*P*
Feeding group			0.704
MF	283 (42.3)	45 (45.0)	
EBB	150 (22.4)	24 (24.0)	
EDB	236 (35.3)	31 (31.0)	
Mother			
Age	30.63±3.74	31.04±4.07	0.312
Education level			0.973
High school or below	84 (12.6)	12 (12.0)	
Junior college	170 (25.4)	24 (24.0)	
Undergraduate	299 (44.7)	45 (45.0)	
Postgraduate or above	116 (17.3)	19 (19.0)	
Occupation			0.364
Employed	492 (73.5)	65 (65.0)	
Self-employed	46 (6.9)	9 (9.0)	
Housewife	100 (14.9)	20 (20.0)	
Other	31 (4.6)	6 (6.0)	
Annual household income			0.898
<150,000	222 (33.3)	31 (31.0)	
150,000–299,999	333 (49.9)	52 (52.0)	
≥300,000	112 (16.8)	17 (17.0)	
Gravidity			0.325
<2 pregnancies	528 (78.9)	74 (74.0)	
≥2 pregnancies	141 (21.1)	26 (26.0)	
Primiparous			0.316
Yes	407 (60.8)	55 (55.0)	
No	262 (39.2)	45 (45.0)	
[a]Pregnancy complications			0.101
Yes	215 (32.1)	41 (41.0)	
No	454 (67.9)	59 (59.0)	
Time to initiation of breastfeeding	1.87±1.63	1.80±1.36	0.671
Infant			0.122
Sex			
Male	347 (51.9)	43 (43.0)	
Female	322 (48.1)	57 (57.0)	
Delivery mode			0.473
Vaginal delivery	372 (55.6)	60 (60.0)	
Cesarean section	297 (44.4)	40 (40.0)	
Birth weight (g)	3342.3±369.8	3345.2±385.1	0.914

Birth length (cm)	50.0±1.22	49.9±1.30	0.772
[b]neonatal diseases			0.208
Yes	624 (93.3)	91 (91.0)	
No	45 (6.7)	9 (9.0)	

Excluded participants (*n*=100) include those lost to follow-up, with missing visit data, incomplete IGSQ, or chronic maternal conditions, among the 769 participants with available baseline data. Ten enrolled pairs without baseline data (1.3% of 779) are not included in this comparison. *P* values from χ^2 test (categorical) or independent t-test (continuous).

Table S6. Effect sizes for baseline comparisons among feeding groups (EDB, EBB, MF)

Variable	EDB vs. EBB	EDB vs. MF	EBB vs. MF
Mothers			
Age (years)	0.111	–0.122	–0.234
Annual household income (≥300k vs. <300k)	–0.240	–0.192	0.049
Gravidity (≥2 vs. <2)	0.406	0.230	–0.176
Primiparous (yes vs. no)	–0.393	–0.299	0.092
Delivery mode (cesarean vs. vaginal)	–0.111	–0.159	–0.048
Pregnancy complications (yes vs. no)	0.003	–0.170	–0.173
Infants			
Sex (male vs. female)	–0.107	–0.032	0.075
Birth weight (g)	–0.0004	0.039	0.038
Birth length (cm)	0.008	0.068	0.055
Neonatal diseases (yes vs. no)	–0.030	0.128	0.157

Values are standardized mean differences (SMDs). For continuous variables (age, birth weight, birth length) and binary variables (all others), SMD quantifies the difference in means between two groups in units of the pooled standard deviation. A positive SMD indicates that the first-named group has a higher mean or proportion than the second-named group; a negative SMD indicates the opposite. An absolute SMD > 0.1 was considered indicative of meaningful imbalance. For time to breastfeeding initiation (non-normally distributed), the overall SMD was 0.404 (P < 0.001); pairwise SMDs are not shown due to non-normality (see Table 1 for medians and IQRs). For multinomial variables (education level, occupation), Cramér's V values were 0.082 and 0.066, respectively (both P > 0.05).

Table S7. Absolute frequency of gastrointestinal symptoms stratified by feeding group (fixed at 1 month) and age

Feeding group	Age	Bloating *n* (%)	Swallowing difficulty *n* (%)	Constipation *n* (%)	Diarrhea *n* (%)	Vomiting *n* (%)
EDB	1m	196 (83.1)	16 (6.8)	25 (10.6)	44 (18.6)	29 (12.3)
	4m	121 (51.3)	15 (6.4)	32 (13.6)	52 (22.0)	30 (12.7)
	6m	91 (38.6)	16 (6.8)	51 (21.6)	50 (21.2)	28 (11.9)
	12m	78 (33.1)	28 (11.9)	100 (42.4)	74 (31.4)	34 (14.4)
EBB	1m	130 (86.7)	20 (13.3)	22 (14.7)	37 (24.7)	27 (18.0)
	4m	82 (54.7)	15 (10.0)	41 (27.3)	51 (34.0)	23 (15.3)
	6m	74 (49.3)	26 (17.3)	49 (32.7)	52 (34.7)	30 (20.0)
	12m	46 (30.7)	24 (16.0)	74 (49.3)	52 (34.7)	27 (18.0)
MF	1m	240 (84.8)	42 (14.8)	57 (20.1)	71 (25.1)	50 (17.7)
	4m	159 (56.2)	32 (11.3)	69 (24.4)	74 (26.1)	50 (17.7)
	6m	129 (45.6)	37 (13.1)	82 (29.0)	83 (29.3)	57 (20.1)
	12m	96 (33.9)	45 (15.9)	142 (50.2)	112 (39.6)	67 (23.7)

Denominators are the baseline total number of infants in each feeding group (EDB=236, EBB=150, MF=283). Missing symptom responses (7 in total, randomly distributed) were treated as “not occurred”.

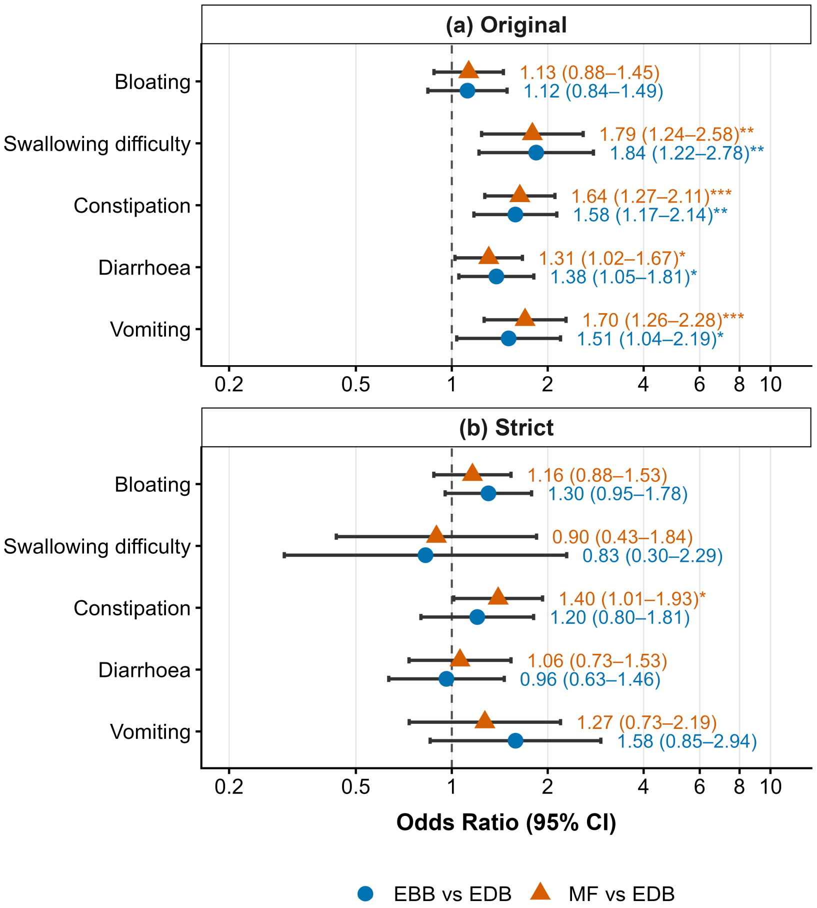

Figure S2. Forest plot comparison of associations between feeding practices and gastrointestinal symptoms using original vs. strict definitions in longitudinal GEE analysis. (a) Original definition (symptoms include "rarely"). (b) Strict definition (only "sometimes", "often", or "almost always" considered as events). Adjusted odds ratios (ORs) and 95% confidence intervals (CIs) for EBB and MF versusEDB are shown. Models adjusted for maternal age, education, occupation, income, gravidity, parity, mode of delivery, pregnancy complications, time to breastfeeding initiation, and infant sex (exchangeable working correlation). Dashed vertical line: OR = 1. Error bars: 95% CI. Blue circles: EBB vs EDB; orange triangles: MF vs EDB. OR and 95% CI values are displayed to the right of each point. *$P < 0.05$, **$P < 0.01$, ***$P < 0.001$.

Table S8a. Longitudinal GEE sensitivity analysis: associations between feeding practices and gastrointestinal symptoms after adjusting for baseline symptoms (4–12 months)

		Main analysis		Sensitivity analysis	
**Symptom**	**Comparison**	**OR (95% CI)**	***P***	**OR (95% CI)**	***P***
Bloating	EBB vs. EDB	1.12 (0.84, 1.49)	0.438	1.37 (1.04, 1.82)	0.028
	MF vs. EDB	1.13 (0.88, 1.45)	0.341	1.31 (1.06, 1.62)	0.014
Swallowing difficulty	EBB vs. EDB	1.84 (1.22, 2.78)	0.004	1.43 (0.87, 2.36)	0.158
	MF vs. EDB	1.79 (1.24, 2.58)	0.002	1.61 (1.10, 2.36)	0.014
Constipation	EBB vs. EDB	1.58 (1.17, 2.14)	0.003	1.11 (0.79, 1.54)	0.557
	MF vs. EDB	1.64 (1.27, 2.11)	<0.001	1.49 (1.16, 1.92)	0.002
Diarrhoea	EBB vs. EDB	1.38 (1.05, 1.81)	0.020	1.20 (0.88, 1.65)	0.248
	MF vs. EDB	1.31 (1.02, 1.67)	0.032	1.10 (0.86, 1.42)	0.449
Vomiting	EBB vs. EDB	1.51 (1.04, 2.19)	0.032	1.00 (0.68, 1.48)	0.998
	MF vs. EDB	1.70 (1.26, 2.28)	<0.001	1.42 (1.07, 1.89)	0.015

Main analysis included all time points (1, 4, 6, 12 months) without adjusting for baseline symptoms. Sensitivity analysis excluded 1-month outcomes and adjusted for the corresponding symptom at 1 month.

Table S8b. Sensitivity analysis adjusting for baseline IGSQ score: comparison of main analysis and sensitivity analysis for IGSQ score

Comparison	Main analysis β (95% CI)	*P*	Sensitivity analysis β (95% CI)	*P*
EBB vs. EDB	0.519 (-0.114, 1.153)	0.108	0.411 (-0.120, 0.942)	0.129
MF vs. EDB	0.950 (0.356, 1.543)	0.002	0.415 (0.002, 0.828)	0.049

Main analysis included all time points (1, 4, 6, 12 months) without adjusting for baseline IGSQ. Sensitivity analysis excluded 1-month outcomes and adjusted for IGSQ score at 1 month.

Table S9a Feeding practices at 1, 4, 6, and 12 months of age

Time point	MF *n* (%)	EBB *n* (%)	EDB *n* (%)	FF *n* (%)	Total N
1 month	283 (42.3)	150 (22.4)	236 (35.3)	0 (0.00)	669
4 months	193 (28.9)	138 (20.7)	311 (46.6)	26 (3.9)	668
6 months	269 (40.2)	136 (20.3)	192 (28.7)	72 (10.8)	669
12 months	151 (22.7)	31 (4.7)	115 (17.3)	368 (55.3)	665

Denominators vary slightly due to missing data at each visit. EDB = exclusive direct breastfeeding; EBB = bottle-fed expressed breastmilk; MF = mixed feeding; FF = exclusive formula feeding. For time-varying sensitivity analyses, FF was reclassified as MF (to maintain three categories consistent with the main analysis and avoid unstable estimates from small cell sizes, e.g., EBB at 12 months, *n*=31).

Table S9b. Sensitivity analysis using time-varying feeding exposure: comparison with main analysis (fixed 1-month exposure)

Model	Comparison	β (95% CI)	*P*
Main analysis<br>(fixed 1-month exposure)	EBB vs. EDB	0.519 (-0.114, 1.153)	0.108
	MF vs. EDB	0.950 (0.356, 1.543)	**0.002**
Time-varying exposure analysis<br>(updated at each visit)	EBB vs. EDB	0.360 (-0.210, 0.930)	0.213
	MF vs. EDB	0.679 (0.207, 1.151)	**0.005**

Main analysis used feeding mode assessed at 1 month of age only. Time-varying analysis used the actual feeding mode reported at 1, 4, 6, and 12 months. Both models were adjusted for the same covariates as Model 3 and used an exchangeable working correlation structure. The time-varying model did not include a feeding mode × time interaction term because the interaction was not significant (P = 0.247); therefore, the reported effects represent average associations over the entire follow-up period.

Table S10a Probiotic use by feeding group and age

Feeding group	1 month	4 months	6 months	12 months
EDB	44/236 (18.6%)	20/236 (8.5%)	16/236 (6.8%)	7/236 (3.0%)
EBB	43/150 (28.7%)	21/150 (14.0%)	16/150 (10.7%)	6/150 (4.0%)
MF	70/283 (24.7%)	45/283 (15.9%)	15/283 (5.3%)	15/283 (5.3%)

Data are presented as n/N (%). Denominators vary slightly due to missing data at each visit. EDB = exclusive direct breastfeeding; EBB = bottle-fed expressed breastmilk; MF = mixed feeding.

Table S10b. Sensitivity analysis adjusting for time-varying probiotic use: comparison with main analysis for IGSQ score (linear GEE)

**Model**	**Comparison**	**β (95% CI)**	***P***
Main (without probiotic adjustment)	EBB vs. EDB	0.519 (-0.114,1.153)	0.108
	MF vs. EDB	0.950 (0.356,1.543)	0.002
Sensitivity (adjusted for probiotic)	EBB vs. EDB	0.472 (-0.163, 1.102)	0.148
	MF vs. EDB	0.943 (0.351, 1.532)	0.002

Main analysis did not adjust for probiotic use. Sensitivity analysis additionally adjusted for time-varying probiotic use (yes/no at each visit). Both models were adjusted for the same covariates as Model 3 (maternal age, education, occupation, income, gravidity, parity, delivery mode, pregnancy complications, time to breastfeeding initiation, infant sex) and used an exchangeable working correlation structure. Values are β coefficients with 95% confidence intervals and P values. EDB = exclusive direct breastfeeding; EBB = bottle-fed expressed breastmilk; MF = mixed feeding.

Table S10c Sensitivity analysis adjusting for time-varying probiotic use: comparison with main analysis for individual gastrointestinal symptoms (logistic GEE)

Symptom	Model	Comparison	OR (95% CI)	*P*
Bloating	Main	EBB vs. EDB	1.12 (0.84, 1.49)	0.438
		MF vs. EDB	1.13 (0.84, 1.45)	0.341
	Adjusted	EBB vs. EDB	1.09 (0.82, 1.45)	0.538
		MF vs. EDB	1.12 (0.87, 1.43)	0.371
Vomiting	Main	EBB vs. EDB	1.51 (1.04, 2.19)	0.032
		MF vs. EDB	1.70 (1.26, 2.28)	<0.001
	Adjusted	EBB vs. EDB	1.46 (1.00, 2.13)	0.048
		MF vs. EDB	1.68 (1.24, 2.27)	0.001
Constipation	Main	EBB vs. EDB	1.58 (1.17, 2.14)	0.003
		MF vs. EDB	1.64 (1.27, 2.11)	<0.001
	Adjusted	EBB vs. EDB	1.57 (1.17, 2.12)	0.003
		MF vs. EDB	1.63 (1.26, 2.10)	<0.001
Diarrhoea	Main	EBB vs. EDB	1.38 (1.05, 1.81)	0.020
		MF vs. EDB	1.31 (1.02, 1.67)	0.032
	Adjusted	EBB vs. EDB	1.35 (1.03, 1.78)	0.032
		MF vs. EDB	1.28 (1.01, 1.63)	0.044
Swallowing difficulty	Main	EBB vs. EDB	1.79 (1.24, 2.58)	0.004
		MF vs. EDB	1.84 (1.22, 2.78)	0.002
	Adjusted	EBB vs. EDB	1.80 (1.19, 2.72)	0.005
		MF vs. EDB	1.78 (1.23, 2.57)	0.002

Main analysis did not adjust for probiotic use. Sensitivity analysis additionally adjusted for time-varying probiotic use (yes/no at each visit). Both models were adjusted for the same covariates as Model 3 (see Supplementary Table F-B) and used an exchangeable working correlation structure. Values are odds ratios (OR) with 95% confidence intervals and *P* values. EDB = exclusive direct breastfeeding; EBB = bottle-fed expressed breastmilk; MF = mixed feeding.
